# Determination of an Extremal in Two-Dimensional Variational Problems Based on the RBF Collocation Method

**DOI:** 10.3390/e24101345

**Published:** 2022-09-23

**Authors:** Ahmad Golbabai, Nima Safaei, Mahboubeh Molavi-Arabshahi

**Affiliations:** School of Mathematics, Iran University of Science and Technology, Narmak, Tehran 16846-13114, Iran

**Keywords:** two-dimensional variational problem, radial basis functions, Lagrange multipliers

## Abstract

This paper introduces a direct method derived from the global radial basis function (RBF) interpolation over arbitrary collocation nodes occurring in variational problems involving functionals that depend on functions of a number of independent variables. This technique parameterizes solutions with an arbitrary RBF and transforms the two-dimensional variational problem (2DVP) into a constrained optimization problem via arbitrary collocation nodes. The advantage of this method lies in its flexibility in selecting between different RBFs for the interpolation and parameterizing a wide range of arbitrary nodal points. Arbitrary collocation points for the center of the RBFs are applied in order to reduce the constrained variation problem into one of a constrained optimization. The Lagrange multiplier technique is used to transform the optimization problem into an algebraic equation system. Three numerical examples indicate the high efficiency and accuracy of the proposed technique.

## 1. Introduction

The purpose of the calculus of variations is to find functionals achieving the extremal (maximum or minimum) value. The direct approaches convert the variational problems (VPs) into a mathematical programming problem. Schechter [1] adopted the direct scheme of Galerkin and Ritz to approximate VPs. The authors of [2,3,4,5,6,7] introduced the Walsh series method, Legendre wavelets technique, Legendre polynomials, Laguerre polynomials, Chebyshev series and differential transformation method, respectively. Yousefi and Dehghan [8] applied the He’s variational iteration technique to simulate variational problems. Recently, Golbabai and Saeedi [9] used a meshless method to approximate VPs arising from a dynamic investment model.

In this paper, we introduce a numerical approach for approximating the two-dimensional variational problem (2DVP) for functionals depending on the function of several independent variables in the following form:

(1) $$J\left[z\left(x,y\right)\right]=\int \int_{\Omega }F\left(x,y,z\left(x,y\right),\frac{\partial }{\partial x}z\left(x,y\right),\frac{\partial }{\partial y}z\left(x,y\right)\right)dxdy,$$

in which *x* and *y* are the independent variables of which z(x,y) is a continuous function with continuous partial derivatives $\frac{\partial }{\partial x}z\left(x,y\right),\frac{\partial }{\partial y}z\left(x,y\right)$ with respect to *x* and *y*, respectively. The symbol $\Omega =\left[x_{\alpha },x_{\beta }\right]\times \left[y_{\alpha },y_{\beta }\right]$ represents the area of the fixed region in the *x*–*y* plane.

In this work, we introduce the radial basis function (RBF) collocation method to simulate 2DVPs for functionals depending on the function of several independent variables. The presented strategy parameterizes the solutions with arbitrary global RBFs (GRBFs) and transforms the 2DVP into a constrained optimization problem by means of arbitrary collocation nodes. One may select the interpolation function from the family of GRBFs, such as the multiquadric (MQ), Gaussian (GA), inverse multiquadric (IMQ), etc., functions. A major benefit of the RBF method is its arbitrary discretization. The proposed technique does not require a node grid for discretizing and provides a higher level of flexibility in selecting the collocation points. By means of the Legendre–Gauss–Lobatto (LGL) quadrature and the method of Lagrange multipliers, the problem is reduced to an algebraic equation system. After solving the algebraic equations, the unknown coefficients can be obtained.

The layout of the current paper is organized as follows: Section 2 introduces some properties of RBFs. Section 3 uses the RBF collocation method to solve variational problems for functionals depending on the function of several independent variables. Section 4 presents three numerical examples illustrating the accuracy of the RBF collocation method. Finally, Section 5 contains the concluding remarks.

## 2. Properties of RBFs

RBFs are very powerful mathematical tools and deserve special attention in the field of computational science. Hardy [10] first introduced the RBFs interpolation to approximate two-dimensional geographical surfaces based on scattered data. Later, Kansa [11,12] first adopted the MQ-RBF collocation technique to approximate PDEs of parabolic, elliptic and hyperbolic types. Recently, Golbabai et al. [13,14] used an RBF collocation technique for a nonlinear models. The meshfree RBF method is used in both local and global forms. Some authors have tried localized-RBF-based strategies, such as the localized-RBF-generated FD (RBF-FD) [15,16,17,18,19,20,21,22,23,24] and the RBF partition of unity (RBF-PU) [25,26,27,28,29,30,31,32,33,34,35].

**Figure 1:**
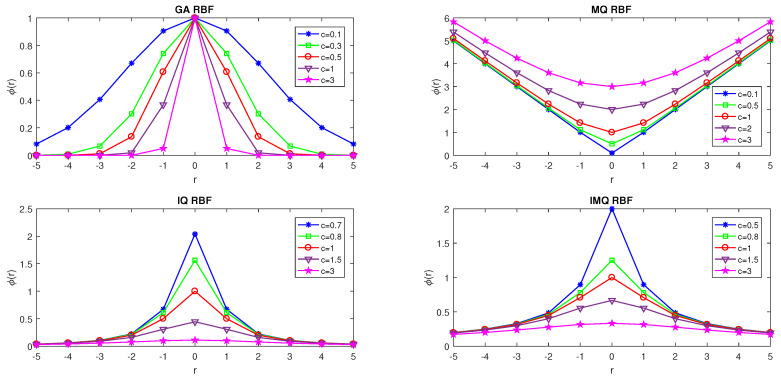
Illustration of some RBFs.

Let $(x_{k},y_{k}),k=1,\ldots ,N$ be a given set of distinct centers. The approximation of a function F(x,y) using RBFs may be written as a linear combination

(2) $$F\left(x,y\right)=\sum_{k=1}^{N}a_{k}\phi_{k}\left(x,y\right)=\sum_{k=1}^{N}a_{k}\phi (∥\left(x,y\right)-\left(x_{k},y_{k}\right)∥),$$

in which $∥\left(x,y\right)-\left(x_{k},y_{k}\right)∥=\sqrt{{\left(x-x_{k}\right)}^{2}+{\left(y-y_{k}\right)}^{2}}$, $\left(x_{k},y_{k}\right)$ are centers and $a_{k}$ are unknown coefficients for k=1,…,N. Table 1 and Figure 1 illustrate some RBFs mathematically and graphically, respectively, where $r=∥\left(x,y\right)-\left(x_{k},y_{k}\right)∥$ and ∥.∥ denote the Euclidean norm and *c* is a positive shape (SP) which controls the width (flatness) of the basis function.

Suppose that $z(x_{k},y_{k}),k=1,\ldots ,N$ is a finite set of distinct nodal points. The unknown coefficients ${\left\{a_{k}\right\}}_{k=1}^{N}$ are determined so that $F\left(x_{k},y_{k}\right)=z_{k}$ for k=1,…,N, which results in the linear system as follows

$$\left[A\right]\left[\begin{array}{c} a_{1}\\ a_{2}\\ \vdots \\ a_{N} \end{array}\right]=\left[\begin{array}{c} z_{1}\\ z_{2}\\ \vdots \\ z_{N} \end{array}\right],$$

where the entries of the matrix A are $A_{ij}=\phi (∥\left(x_{i},y_{i}\right)-\left(x_{j},y_{j}\right)∥),i,j=1,\ldots ,N$ [36,37]. Since we choose ϕ to have global support, this method causes a dense matrix A. The matrix A has been proven to be positive definite for distinct interpolation nodes. Micchelli [38] proved that the matrix A was positive definite for GA, IMQ and TPS RBF and conditionally positive definite for the MQ RBF method.

### 2.1. SP Strategies

The selection of the SP has a considerable effect on the stability and accuracy of an RBF method. There are different methods to select SPs. The most common are computing errors with distinct SPs and selecting the best one. SPs are categorized into two classes: constant and variable SPs.

#### 2.1.1. Constant SPs

Constant SPs can be considered by Hardy and Franke’s formulas as follows:

- Hardy’s SP [10]
  (3) $$c=\frac{1}{0.815d},\;d=\frac{1}{N}\sum_{i=1}^{N}d_{i},$$
  in which *N* and $d_{i}$ represent the total number of centers and the distance from the *i*th center to the nearest neighbor, respectively.
- Franke’s SP [39]
  (4) $$c=\frac{\sqrt{N}}{1.25D},$$
  in which *N* and *D* denote the total number of centers and the diameter of the smallest circle encompassing all the center locations, respectively.

#### 2.1.2. Variable SPs

A variable SP approach utilizes a different SP value at every center. This facilitates obtaining a different entry in the RBF matrices, leading to a lower condition number. Here, we list a number of variable SPs:

- Exponentially SP (ESP) [11]
  (5) $$c_{j}={\left(c_{\min}^{2}{\left(\frac{c_{\max}^{2}}{c_{\min}^{2}}\right)}^{\frac{j-1}{N-1}}\right)}^{\frac{1}{2}},\;j=1,\ldots ,N$$
- Sinusoidal SP (SSP) [40]
  (6) $$c_{j}=c_{\min}+\left(c_{\max}-c_{\min}\right)\sin\left(\frac{(j-1)\pi }{2(N-1)}\right),\;j=1,\ldots ,N$$
- Linear SP (LSP) [12]
  (7) $$c_{j}=c_{\min}+\left(\frac{c_{\max}-c_{\min}}{N}\right)j,\;j=1,\ldots ,N$$
- Random SP (RSP) [41]
  (8) $$c_{j}=c_{\min}+\left(\frac{c_{\max}-c_{\min}}{N}\right)rand\left(1,N\right),\;j=1,\ldots ,N$$
  where $c_{\max}$ and $c_{\min}$ represent the maximum and minimum of $c_{j}$’s, respectively.

## 3. Numerical Solution of the Model

This section uses the RBF collocation technique to simulate variational problems for functionals depending on a function of several independent variables based on interpolating (GRBFs) over arbitrary collocation nodes. Let us find the extremal values of the following functional,

(9) $$J\left[z\left(x,y\right)\right]=\underset{\Omega }{\;\int \int \;}F\left(x,y,z\left(x,y\right),\frac{\partial }{\partial x}z\left(x,y\right),\frac{\partial }{\partial y}z\left(x,y\right)\right)dxdy,$$

with the given boundary conditions (BCs) of the form

(10) z(x,y)=g(x,y),(x,y)∈∂Ω.

In order to provide a framework with a higher flexibility, different groups of collocation points, containing nodes with equal and unequal spacing, could be arbitrarily selected for the discretization. For instance, a set of Chebyshev–Gauss (CG), Gauss–Legendre (GL), Gauss–Lobatto (GLO), Gauss–Laguerre (GLA), Legendre–Gauss–Lobatto (LGL) and Chebyshev–Gauss–Lobatto (CGL) points can be chosen as a set of unequally spaced orthogonal points to approximate the desired model [42,43]. Now, we consider the following 2DVP for functionals depending on the function of several independent variables of Equations (9) and (10). The solution z(x,y) is approximated using RBFs as

(11) $$z\left(x,y\right)\approx z^{N,M}\left(x,y\right)=\sum_{i=1}^{N}\sum_{j=1}^{M}a_{ij}\phi (∥\left(x,y\right)-\left(x_{i},y_{i}\right)∥)=\sum_{i=1}^{N}\sum_{j=1}^{M}a_{ij}\phi_{ij}\left(x,y\right),$$

where $z^{N,M}\left(x,y\right)$ denotes the RBF interpolation of z(x,y). Furthermore, $\phi_{ij}\left(x,y\right)=\phi (∥\left(x,y\right)-\left(x_{i},y_{j}\right)∥)$ denotes an RBF and $a_{ij}$ represent the RBF weights related to $z^{N,M}\left(x,y\right)$.

**Theorem** **1.** *Let $z^{N,M}\left(x,y\right)=\sum_{i=1}^{N}\sum_{j=1}^{M}a_{ij}\phi_{ij}\left(x,y\right)$ and $\phi \left(r\right)=e^{-cr^{2}}$, then there exist partial derivatives with respect to *x* and *y* as follows:*

(12) $$\frac{\partial }{\partial x}z^{N,M}\left(x,y\right)=\sum_{i=1}^{N}\sum_{j=1}^{M}-2ca_{ij}\left(x-x_{i}\right)\phi_{ij}\left(x,y\right),$$

(13) $$\frac{\partial }{\partial y}z^{N,M}\left(x,y\right)=\sum_{i=1}^{N}\sum_{j=1}^{M}-2ca_{ij}\left(y-y_{j}\right)\phi_{ij}\left(x,y\right).$$

**Proof.**  According to the definition of RBFs, $r=∥\left(x,y\right)-\left(x_{i},y_{j}\right)∥={\left({\left(x-x_{i}\right)}^{2}+{\left(y-y_{j}\right)}^{2}\right)}^{\frac{1}{2}}$, then the chain rule implies

$$\frac{\partial }{\partial x}\phi \left(r\right)=\frac{d}{dr}\phi \left(r\right)\frac{\partial }{\partial x}r\left(x,y\right)=-2cr\phi \left(r\right)\frac{\left(x-x_{i}\right)}{{\left({\left(x-x_{i}\right)}^{2}+{\left(y-y_{j}\right)}^{2}\right)}^{\frac{1}{2}}}$$

$$=-2c\left(x-x_{i}\right)\phi \left(r\right).$$

Now, we obtain the partial derivative of $z^{N,M}\left(x,y\right)$ with respect to *x*,

$$\frac{\partial }{\partial x}z^{N,M}\left(x,y\right)=\sum_{i=1}^{N}\sum_{j=1}^{M}a_{ij}\frac{\partial }{\partial x}\phi_{ij}\left(x,y\right)=\sum_{i=1}^{N}\sum_{j=1}^{M}-2ca_{ij}\left(x-x_{i}\right)\phi_{ij}\left(x,y\right).$$

Subsequently, we can obtain the partial derivative of $z^{N,M}\left(x,y\right)$ with respect to *y*. □

**Theorem** **2.** *Let $z^{N,M}\left(x,y\right)=\sum_{i=1}^{N}\sum_{j=1}^{M}a_{ij}\phi_{ij}\left(x,y\right)$ and $\phi \left(r\right)=\sqrt{c^{2}+r^{2}}$, then there exist partial derivatives with respect to *x* and *y* as follows:*

(14) $$\frac{\partial }{\partial x}z^{N,M}\left(x,y\right)=\sum_{i=1}^{N}\sum_{j=1}^{M}a_{ij}\left(x-x_{i}\right){\left(\phi_{ij}\left(x,y\right)\right)}^{-1},$$

(15) $$\frac{\partial }{\partial y}z^{N,M}\left(x,y\right)=\sum_{i=1}^{N}\sum_{j=1}^{M}a_{ij}\left(y-y_{j}\right){\left(\phi_{ij}\left(x,y\right)\right)}^{-1}.$$

**Proof.**  According to the definition of RBFs, $r=∥\left(x,y\right)-\left(x_{i},y_{j}\right)∥={\left({\left(x-x_{i}\right)}^{2}+{\left(y-y_{j}\right)}^{2}\right)}^{\frac{1}{2}}$. Then, the chain rule implies

$$\frac{\partial }{\partial x}\phi \left(r\right)=\frac{d}{dr}\phi \left(r\right)\frac{\partial }{\partial x}r\left(x,y\right)=r{\left(\phi \left(r\right)\right)}^{-1}\frac{\left(x-x_{i}\right)}{{\left({\left(x-x_{i}\right)}^{2}+{\left(y-y_{j}\right)}^{2}\right)}^{\frac{1}{2}}}$$

$$=\left(x-x_{i}\right){\left(\phi \left(r\right)\right)}^{-1}.$$

Now, we obtain the partial derivative of $z^{N,M}\left(x,y\right)$ with respect to *x*,

$$\frac{\partial }{\partial x}z^{N,M}\left(x,y\right)=\sum_{i=1}^{N}\sum_{j=1}^{M}a_{ij}\frac{\partial }{\partial x}\phi_{ij}\left(x,y\right)=\sum_{i=1}^{N}\sum_{j=1}^{M}a_{ij}\left(x-x_{i}\right){\left(\phi_{ij}\left(x,y\right)\right)}^{-1}.$$

Correspondingly, we can achieve the partial derivative of $z^{N,M}\left(x,y\right)$ with respect to *y*.  □

Now, by substituting Equation (11) and the partial derivatives obtained from Theorem 1 or Theorem 2 in Equations (9) and (10), we have

(16) $$\int_{y_{\alpha }}^{y_{\beta }}\int_{x_{\alpha }}^{x_{\beta }}F(x,y,z\left(x,y\right),\frac{\partial }{\partial x}z\left(x,y\right),\frac{\partial }{\partial y}z\left(x,y\right))dxdy=\int_{y_{\alpha }}^{y_{\beta }}\int_{x_{\alpha }}^{x_{\beta }}F(x,y,\sum_{i=1}^{N}\sum_{j=1}^{M}a_{ij}$$

$$\phi_{ij}\left(x,y\right),\sum_{i=1}^{N}\sum_{j=1}^{M}a_{ij}\frac{\partial }{\partial x}\phi_{ij}\left(x,y\right),\sum_{i=1}^{N}\sum_{j=1}^{M}a_{ij}\frac{\partial }{\partial y}\phi_{ij}\left(x,y\right))dxdy.$$

(17) $$\sum_{i=1}^{N}\sum_{j=1}^{M}a_{ij}\phi_{ij}\left(x,y\right)=g\left(x,y\right),\;\left(x,y\right)\in \partial \Omega$$

By applying LGL quadrature, we can approximate Equation (16) as follows,

(18) $$\int_{y_{\alpha }}^{y_{\beta }}\int_{x_{\alpha }}^{x_{\beta }}F(x,y,\sum_{i=1}^{N}\sum_{j=1}^{M}a_{ij}\phi_{ij}\left(x,y\right),\sum_{i=1}^{N}\sum_{j=1}^{M}a_{ij}\frac{\partial }{\partial x}\phi_{ij}\left(x,y\right),\sum_{i=1}^{N}\sum_{j=1}^{M}a_{ij}\frac{\partial }{\partial y}\phi_{ij}\left(x,y\right))dxdy,$$

$$=\frac{\left(y_{\beta }-y_{\alpha }\right)}{2}\frac{\left(x_{\beta }-x_{\alpha }\right)}{2}\sum_{k_{1}=1}^{N}\sum_{k_{2}=1}^{M}F(x_{k_{1}},y_{k_{2}},\sum_{i=1}^{N}\sum_{j=1}^{M}a_{ij}\phi_{ij}\left(x_{k_{1}},y_{k_{2}}\right),\sum_{i=1}^{N}\sum_{j=1}^{M}a_{ij}\frac{\partial }{\partial x}\phi_{ij}\left(x_{k_{1}},y_{k_{2}}\right),$$

$$\sum_{i=1}^{N}\sum_{j=1}^{M}a_{ij}\frac{\partial }{\partial y}\phi_{ij}\left(x_{k_{1}},y_{k_{2}}\right)),$$

in which $x_{k_{1}}=\frac{x_{\beta }-x_{\alpha }}{2}t_{k}+\frac{x_{\beta }+x_{\alpha }}{2}$ and $y_{k_{2}}=\frac{y_{\beta }-y_{\alpha }}{2}t_{k}+\frac{y_{\beta }+y_{\alpha }}{2}$, $t_{k}$ denote the nodes LGL and $w_{k_{1}}$, $w_{k_{2}}$ represent the LGL weights associated with LGL nodes $t_{k}\in \left[-1,1\right]$, described as

$$w_{k}=\frac{2}{\left(N-1\right)N{\left(P_{N-1}\left(t_{k}\right)\right)}^{2}}\;k=1,\ldots ,M,$$

in which $P_{N-1}$ denotes a Legendre polynomial of degree N−1 [44] and $x_{i}$ represent the RBF centers.

Finally, the constrained variational problem for functionals depending on the function of several independent variables of Equations (9) and (10) reduces to a constrained optimization problem

(19) $$J\left(a\right)=\frac{\left(y_{\beta }-y_{\alpha }\right)}{2}\frac{\left(x_{\beta }-x_{\alpha }\right)}{2}\sum_{k_{1}=1}^{N}\sum_{k_{2}=1}^{M}F(x_{k_{1}},y_{k_{2}},\sum_{i=1}^{N}\sum_{j=1}^{M}a_{ij}\phi_{ij}\left(x_{k_{1}},y_{k_{2}}\right),\sum_{i=1}^{N}\sum_{j=1}^{M}a_{ij}$$

$$\frac{\partial }{\partial x}\phi_{ij}\left(x_{k_{1}},y_{k_{2}}\right),\sum_{i=1}^{N}\sum_{j=1}^{M}a_{ij}\frac{\partial }{\partial y}\phi_{ij}\left(x_{k_{1}},y_{k_{2}}\right)),$$

subject to

(20) $$\sum_{i=1}^{N}\sum_{j=1}^{M}a_{ij}\phi_{ij}\left(x,y\right)=g\left(x,y\right),\;\left(x,y\right)\in \partial \Omega .$$

To solve the optimization problem of Equations (19) and (20), we adopt a Lagrange multipliers scheme and transform the problem into the following unconstrained optimization problem, so that,

(21) $$J^{*}\left(a\right)=J\left(a\right)+\lambda \left(\sum_{i=1}^{N}\sum_{j=1}^{M}a_{ij}\phi_{ij}\left(x,y\right)-g\left(x,y\right)\right).$$

where λ is the Lagrange multiplier related to the BCs. The unknown coefficients $a_{ij},i=1,\ldots ,N,j=1,\ldots ,M$ can be determined after solving the system as follows:

$$\frac{\partial J^{*}\left(a\right)}{\partial a_{ij}}=0,i=1,\ldots ,N,j=1,\ldots ,M,$$

$$\frac{\partial J^{*}\left(a\right)}{\partial \lambda }=0.$$

## 4. Numerical Experiments

**Figure 2:**
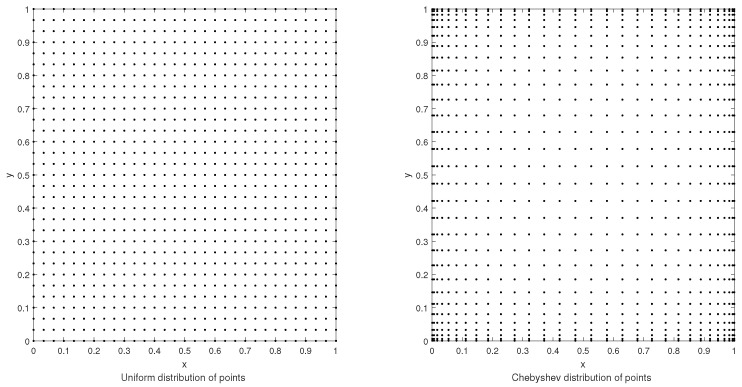
Illustration of the uniformly distributed and Chebyshev nodes.

This section considers three 2DVPs involving functionals that depend on functions of more than one independent variable to demonstrate the effectiveness of the presented technique. The numerical results indicate the advantages of this approach over the upwind technique, variational iteration scheme and other numerical techniques. In three test problems, different values of the SP and the data centers including uniform and Chebyshev nodes were adopted to show the advantage of the proposed strategy. For this aim, we defined the $L_{\infty }$ and $L_{\mathrm{rms}}$ norm errors to assess the efficiency and accuracy as:

$$\begin{array}{cc} \; & L_{\infty }=\max_{1\leq i,j\leq N,M}|z\left(x_{i},y_{j}\right)-z^{N,M}\left(x_{i},y_{j}\right)|,\\ \; & L_{\mathrm{rms}}=\sqrt{\frac{1}{N+M}\sum_{i=1}^{N}\sum_{j=1}^{M}{\left(z\left(x_{i},y_{j}\right)-z^{N,M}\left(x_{i},y_{j}\right)\right)}^{2}}, \end{array}$$

where $z(x_{i},y_{j})$ and $z^{N,M}\left(x_{i},y_{j}\right)$ represent the exact and approximate solutions, respectively. Figure 2 shows the uniformly distributed and Chebyshev nodes. All numerical simulations were done by using MATLAB R2013b on a computer system having a configuration with 4.00 GB of RAM.

**Example** **1.** *Let us consider the following 2DVP of finding the extremal of the functional*

$$J\left[z\left(x,y\right)\right]=\int_{0}^{1}\int_{0}^{1}\left({\left(\frac{\partial z}{\partial x}\right)}^{2}-{\left(\frac{\partial z}{\partial y}\right)}^{2}\right)dxdy,$$

*with the BCs*

z(x,0)=sin(πx),z(0,y)=0,z(1,y)=0.

*The exact solution of this problem is z(x,y)=sin(πx)cos(πy).*

**Figure 3:**
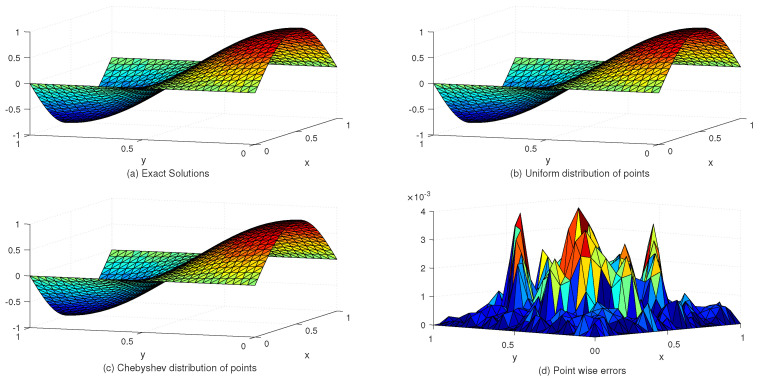
The behavior of exact and approximate solutions as well as numerical errors with $c=10^{-2}$ and N=31×31 in Example 1.

**Figure 4:**
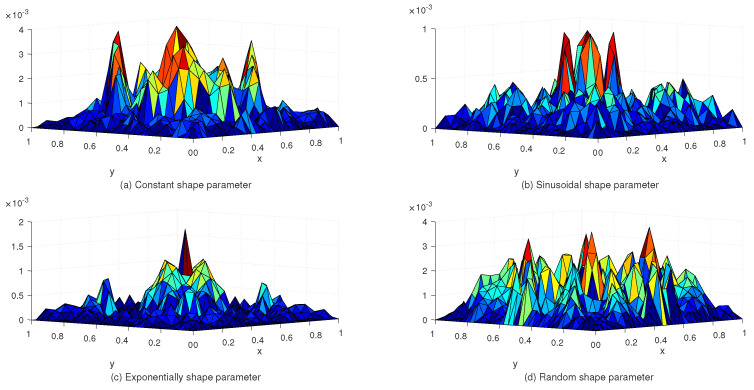
The behavior of numerical errors for constant and variable SPs with N=31×31 in Example 1.

Table 2 lists the $L_{\infty }$ and $L_{\mathrm{rms}}$ error norms for several values of *c* with the uniform and Chebyshev nodes. In view of Table 2, we see that the accuracy of the numerical solution for uniform nodes is significantly better than that for Chebyshev nodes. Moreover, Table 3 shows the exact and approximate values of $z^{4,4}\left(x,y\right)$ based on the proposed method. Figure 3 displays the exact and approximate solutions as well as the numerical errors with uniform and Chebyshev nodes at $c=10^{-2}$ and $N_{x}\times N_{y}=31\times 31$. Finally, Figure 4 represents the behavior of the numerical errors for constant and variable SPs.

**Example** **2.** *Consider the following 2DVP*

$$J\left[z\left(x,y\right)\right]=\int_{0}^{1}\int_{0}^{1}-2z^{2}+z\frac{\partial z}{\partial x}+\frac{1}{2}{\left(\frac{\partial z}{\partial y}\right)}^{2}dxdy,$$

*with the BCs*

z(x,0)=0,z(0,y)=0,z(1,y)=0,

*so that exact solution is as:*

$$z\left(x,y\right)=\sin\left(\frac{\pi }{2}x\right)\sin\left(2y\right).$$

**Figure 5:**
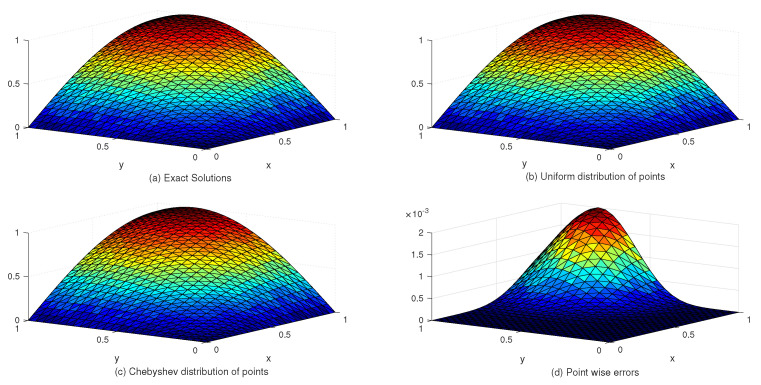
The behavior of exact and approximate solutions as well as numerical errors with $c=10^{-2}$, N=25×25 in Example 2.

**Figure 6:**
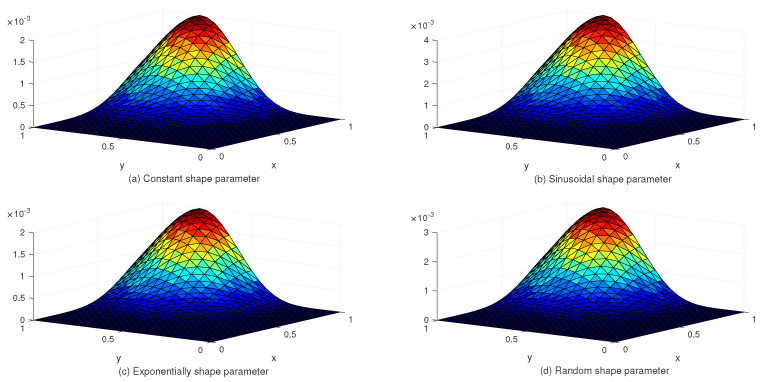
The behavior of numerical errors for constant and variable SPs with N=31×31 in Example 2.

Table 4 compares the $L_{\infty }$ and $L_{\mathrm{rms}}$ error norms with uniform and Chebyshev nodes at different values of *c* and global data centers. Figure 5 shows the exact and approximate solutions as well as the numerical errors with uniform and Chebyshev nodes at $c=10^{-2}$, $N_{x}\times N_{y}=25\times 25$. Finally, Figure 6 displays the behavior of the numerical errors for constant and variable SPs.

**Example** **3.** *Finally, we consider the following 2DVP*

$$J\left[z\left(x,y\right)\right]=\int_{0}^{1}\int_{0}^{1}z^{2}+{\left(\frac{\partial z}{\partial x}\right)}^{2}+\frac{\partial z}{\partial x}\frac{\partial z}{\partial y}+{\left(\frac{\partial z}{\partial y}\right)}^{2}dxdy,$$

*with the BCs*

$$z\left(x,0\right)=e^{-x},z\left(0,y\right)=e^{y},z\left(1,y\right)=e^{y-1},$$

*so that the exact solution has the following form:*

$$z\left(x,y\right)=e^{-x}e^{y}.$$

**Figure 7:**
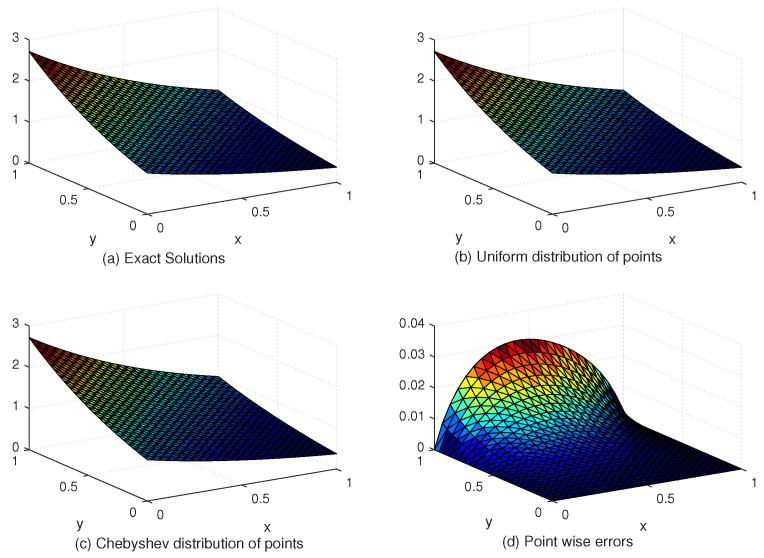
The behavior of exact and approximate solutions as well as numerical errors with $c=10^{-2}$ and N=31×31 in Example 3.

**Figure 8:**
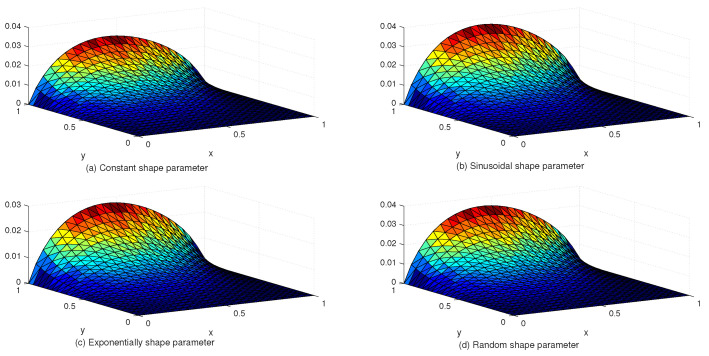
The behavior of numerical errors for constant and variable SPs with N=31×31 in Example 3.

Table 5 compares the $L_{\infty }$ and $L_{\mathrm{rms}}$ error norms with uniform and Chebyshev nodes at different values of SPs, *c* and global data centers. Figure 7 shows the exact and approximate solutions as well as the numerical errors with uniform and Chebyshev nodes at $c=10^{-2}$, $N_{x}\times N_{y}=31\times 31$. Finally, Figure 8 displays the behavior of the numerical errors for constant and variable SPs with $N_{x}\times N_{y}=31\times 31$.

## 5. Final Remarks

Variational problems with multiple integrals occur in various applications. This paper studied the RBF collocation scheme to solve the 2DVP containing functionals that depend on the function of more than one independent variable. Moreover, this method was extended to problems of higher dimensions. The main aim of this work was to present an RBF collocation technique that did not need mesh generation to estimate the solution of 2DVP. Combining the RBF collocation technique with the Legendre–Gauss–Lobatto quadrature reduces the 2DVP to an algebraic equation system. A variable shape parameter approach was introduced for the accuracy and stability of the RBF technique. Finally, numerical experiments validated the efficiency of the presented method.

## Figures and Tables

**Table 1:** Mathematical form of some RBFs, $(r=∥\left(x,y\right)-\left(x_{i},y_{j}\right)∥,c>0$).

Name	ϕ(r)
Inverse quadratic (IQ)	$\phi \left(r\right)=\frac{1}{r^{2}+c^{2}}$
Multiquadric (MQ)	$\phi \left(r\right)={\left(c^{2}+r^{2}\right)}^{1/2}$
Gaussian (GA)	$\phi \left(r\right)=e^{-cr^{2}}$
Inverse multiquadric (IMQ)	$\phi \left(r\right)={\left(c^{2}+r^{2}\right)}^{-1/2}$

**Table 2:** The $L_{\infty }$ and $L_{\mathrm{rms}}$ error norms for several values of *c* with the uniform and Chebyshev nodes in Example (1).

	$N_{x}\times N_{y}$	Uniform Nodes		Chebyshev Nodes	
		9×9	19×19	9×9	19×19
$c=10^{-3}$	$L_{\infty }$	5.2139 × 10${}^{-2}$	1.6732 × 10${}^{-2}$	2.7502 × 10${}^{-1}$	9.2447 × 10${}^{-2}$
	$L_{\mathrm{rms}}$	1.7494 × 10${}^{-2}$	5.6905 × 10${}^{-3}$	6.2467 × 10${}^{-2}$	2.3076 × 10${}^{-2}$
$c=10^{-2}$	$L_{\infty }$	3.4685 × 10${}^{-2}$	8.1158 × 10${}^{-3}$	1.0786 × 10${}^{-1}$	3.3077 × 10${}^{-1}$
	$L_{\mathrm{rms}}$	1.1058 × 10${}^{-2}$	2.1701 × 10${}^{-3}$	2.9463 × 10${}^{-2}$	5.7194 × 10${}^{-2}$
$c=10^{-1}$	$L_{\infty }$	1.2334 × 10${}^{-2}$	3.2135 × 10${}^{-2}$	3.7442 × 10${}^{-2}$	7.4346 × 10${}^{-3}$
	$L_{\mathrm{rms}}$	4.0420 × 10${}^{-3}$	5.7063 × 10${}^{-3}$	9.5542 × 10${}^{-3}$	2.2650 × 10${}^{-3}$

**Table 3:** The approximate and exact values of $z^{4,4}\left(x,y\right)$.

*x*	*y*	Exact Solution	Approximate Solution
0.00	0.00	0.000000000000000	1.637090463191271$\;\times \;10^{-11}$
	0.25	0.000000000000000	4.547473508864641$\;\times \;10^{-12}$
	0.75	0.000000000000000	0.000000000000000
	1.00	0.000000000000000	5.456968210637569$\;\times \;10^{-12}$
0.25	0.00	0.707106781186548	0.707106781194852
	0.25	0.500000000000000	0.501532990779197
	0.75	−0.500000000000000	−0.497730229812078
	1.00	−0.707106781186548	−0.707120813167421
0.75	0.00	0.707106781186548	0.707106781186667
	0.25	0.500000000000000	0.501530239744170
	0.75	−0.500000000000000	−0.497733125168452
	1.00	−0.707106781186548	−0.707092753664256
1.00	0.00	1.224646799147353$\;\times \;10^{-16}$	9.094947017729282$\;\times \;10^{-12}$
	0.25	8.659560562354929$\;\times \;10^{-17}$	0.000000000000000
	0.75	−8.659560562354932$\;\times \;10^{-17}$	0.000000000000000
	1.00	−1.224646799147353$\;\times \;10^{-16}$	−3.637978807091713$\;\times \;10^{-12}$

**Table 4:** The $L_{\infty }$ and $L_{\mathrm{rms}}$ error norms for several values of *c* with the uniform and Chebyshev nodes in Example 2.

	$N_{x}\times N_{y}$	Uniform Nodes		Chebyshev Nodes	
		9×9	19×19	9×9	19×19
$c=10^{-3}$	$L_{\infty }$	2.7079 × 10${}^{-2}$	9.1132 × 10${}^{-3}$	5.8468 × 10${}^{-2}$	2.3114 × 10${}^{-2}$
	$L_{\mathrm{rms}}$	9.6056 × 10${}^{-3}$	3.0467 × 10${}^{-3}$	2.1192 × 10${}^{-2}$	7.9968 × 10${}^{-3}$
$c=10^{-2}$	$L_{\infty }$	2.6386 × 10${}^{-2}$	2.0016 × 10${}^{-2}$	3.7585 × 10${}^{-2}$	2.6196 × 10${}^{-2}$
	$L_{\mathrm{rms}}$	8.7035 × 10${}^{-3}$	3.7800 × 10${}^{-3}$	1.4882 × 10${}^{-2}$	5.4219 × 10${}^{-3}$
$c=10^{-1}$	$L_{\infty }$	5.5040 × 10${}^{-2}$	7.0839 × 10${}^{-2}$	4.1096 × 10${}^{-3}$	8.9954 × 10${}^{-3}$
	$L_{\mathrm{rms}}$	1.4043 × 10${}^{-2}$	1.5971 × 10${}^{-2}$	1.1637 × 10${}^{-3}$	2.5894 × 10${}^{-3}$

**Table 5:** The $L_{\infty }$ and $L_{\mathrm{rms}}$ error norms for several values of *c* with the uniform and Chebyshev nodes in Example 3.

	$N_{x}\times N_{y}$	Uniform Nodes		Chebyshev Nodes	
		9×9	19×19	9×9	19×19
$c=10^{-3}$	$L_{\infty }$	3.0765 × 10${}^{-1}$	3.0979 × 10${}^{-1}$	3.2145 × 10${}^{-1}$	3.1490 × 10${}^{-1}$
	$L_{\mathrm{rms}}$	1.0002 × 10${}^{-1}$	9.5446 × 10${}^{-2}$	1.0244 × 10${}^{-1}$	9.8804 × 10${}^{-2}$
$c=10^{-2}$	$L_{\infty }$	3.0461 × 10${}^{-1}$	3.0830 × 10${}^{-1}$	3.1653 × 10${}^{-1}$	3.1073 × 10${}^{-1}$
	$L_{\mathrm{rms}}$	9.8675 × 10${}^{-2}$	9.4906 × 10${}^{-2}$	1.0027 × 10${}^{-1}$	9.7411 × 10${}^{-2}$
$c=10^{-1}$	$L_{\infty }$	3.0142 × 10${}^{-1}$	3.0895 × 10${}^{-1}$	3.0630 × 10${}^{-1}$	3.0885 × 10${}^{-1}$
	$L_{\mathrm{rms}}$	9.7024 × 10${}^{-2}$	9.4884 × 10${}^{-2}$	9.7018 × 10${}^{-2}$	9.6816 × 10${}^{-2}$

## Data Availability

Not Applicable.
